# Efficacy observation of electromyography-guided targeted injection of swallowing muscles for treating dysphagia resulting from medullary paralysis

**DOI:** 10.3389/fneur.2026.1714495

**Published:** 2026-05-29

**Authors:** Hong Ren, Yan Shang, Tiantian Wang, Chunxiang Zhao, Pei Wang, Yue Yang, Yufei Yin

**Affiliations:** 1 Department of 1^st^ Rehabilitation Medicine, Baoding No.1 Central Hospital, Baoding, Hebei, China; 2 Department of Encephalopathy, The Traditional Chinese Medicine Hospital of Ding Zhou, Dingzhou, Hebei, China; 3 Department of 3^rd^ Neurology, Baoding No.1 Central Hospital, Baoding, Hebei, China

**Keywords:** dysphagia, efficacy, electromyography-guided, medullary paralysis, videofluoroscopic swallowing study

## Abstract

**Objective:**

To observe the clinical efficacy of electromyography (EMG)-guided targeted mecobalamin injections for treating dysphagia resulting from medullary paralysis and to investigate effective dysphagia management strategies.

**Methods:**

This study was a prospective randomized controlled trial. A total of 110 patients with dysphagia due to post-stroke bulbar palsy were enrolled at Baoding No.1 Central Hospital from February 2017 to December 2020. Patients were randomly assigned using a random number table to either a control group (*n* = 55) receiving conventional pharmacotherapy combined with rehabilitation training, or a treatment group (*n* = 55) receiving the same conventional therapy plus additional EMG-guided targeted injections of mecobalamin into the swallowing muscles. Swallowing function was assessed using the Wada water swallowing test (WST) and videofluoroscopic swallowing study (VFSS) after 2 weeks of treatment.

**Results:**

The treatment group showed a significantly higher overall response rate on the WST than the control group (*P* < 0.05). Based on VFSS, the marked and overall effectiveness rates were 50.9% and 96.4% in the treatment group, respectively, significantly higher than the corresponding rates of 18.2% and 83.6% in the control group (both *P* < 0.05). The incidence of aspiration decreased significantly in both groups post-treatment (*P* < 0.05), with a more pronounced reduction observed in the treatment group (*P* < 0.05).

**Conclusion:**

EMG-guided targeted injection of mecobalamin into swallowing muscles is an effective adjunctive strategy for enhancing swallowing function in patients with dysphagia due to post-stroke medullary paralysis.

## Introduction

Swallowing is a highly coordinated process orchestrated by the medullary swallowing center (1, 2). Medullary paralysis, caused by medullary infarction or injury, directly impairs this center, leading to oropharyngeal dysphagia. This condition manifests as difficulty eating, choking while drinking, articulation disorders, and hoarseness (3), and can result in serious complications such as malnutrition, aspiration pneumonia, and even suffocation. Current conventional treatments, including rehabilitation training, neuromuscular electrical stimulation, and acupuncture, often have slow onset and suboptimal efficacy (4).

Mecobalamin, an active form of vitamin B12, participates in methyl transfer reactions, promoting neuronal cell metabolism, myelination, and synaptic development, thereby facilitating axonal regeneration and neuromotor functional recovery (5). This study aims to explore whether precise injection of mecobalamin into affected swallowing muscles under EMG guidance can accelerate swallowing recovery through local neurotrophic support. To objectively evaluate treatment outcomes, we employed videofluoroscopic swallowing study (VFSS), the gold standard for assessing pharyngeal phase dysphagia, in conjunction with the bedside water swallowing test (WST) (6).

## Materials and methods

This study was a prospective randomized controlled trial. A total of 110 patients with dysphagia caused by medullary infarction were selected from the Department of Rehabilitation Medicine and Neurology at Baoding No. 1 Central Hospital between February 2017 and December 2020. Utilizing a random number table, patients were randomly assigned to either the treatment group or the control group in a 1:1 ratio, with 55 patients in each group. The study was approved by the Institutional Ethics Committee of Baoding No.1 Central Hospital (No.: [2019]084; date: November 18, 2019) and was registered with the Chinese Clinical Trial Registry (Registration No.: ChiCTR-ROC-17013962). Written informed consent was obtained from all participants.

Inclusion criteria: (i) Diagnosis of cerebrovascular disease according to the Chinese Neuroscience Society's “Diagnostic criteria of cerebrovascular diseases in China” (7), with MRI-confirmed medullary lesion and presenting with dysphagia; (ii) Wada water swallowing test (WST) score ≥ level 3 and VFSS-confirmed pharyngeal phase dysphagia; (iii) Age 20–70 years, clear consciousness, stable vital signs, and ability to cooperate with examination; (iv) No other comorbidities limiting activity.

Exclusion criteria: (i) Major organ dysfunction (cardiac, pulmonary, or renal failure); (ii) Disease progression, new cerebral infarction, or cerebral hemorrhage; (iii) Dysphagia from other etiologies; (iv) History of iodine allergy; (v) Severe cognitive impairment or psychiatric abnormalities precluding cooperation; (vi) Non-adherence to the study protocol.

### Treatment methods

Control group: Patients received: (1) Conventional neurological pharmacotherapy (antihypertensives, lipid-lowering agents, glucose control drugs, brain metabolism enhancers, and neuroprotective drugs), along with comprehensive hemiplegic limb training. (2) Local low-frequency electrical stimulation (Shijiazhuang Dukang Medical Equipment Co., Ltd., Hebei Medical Device Registration Approval No.: 20142260328) with electrodes placed bilaterally on the larynx, with an intensity setting of 2–5, for 20 min daily. (3) Structured rehabilitation training including oral motor exercises, Shaker exercises, and Mendelsohn maneuvers, supplemented by menthol ice pops stimulation (8). All sessions were conducted once daily in the morning by the same therapist, followed by family-guided practice in the afternoon, each session lasting 30 min.

**Figure 1:**
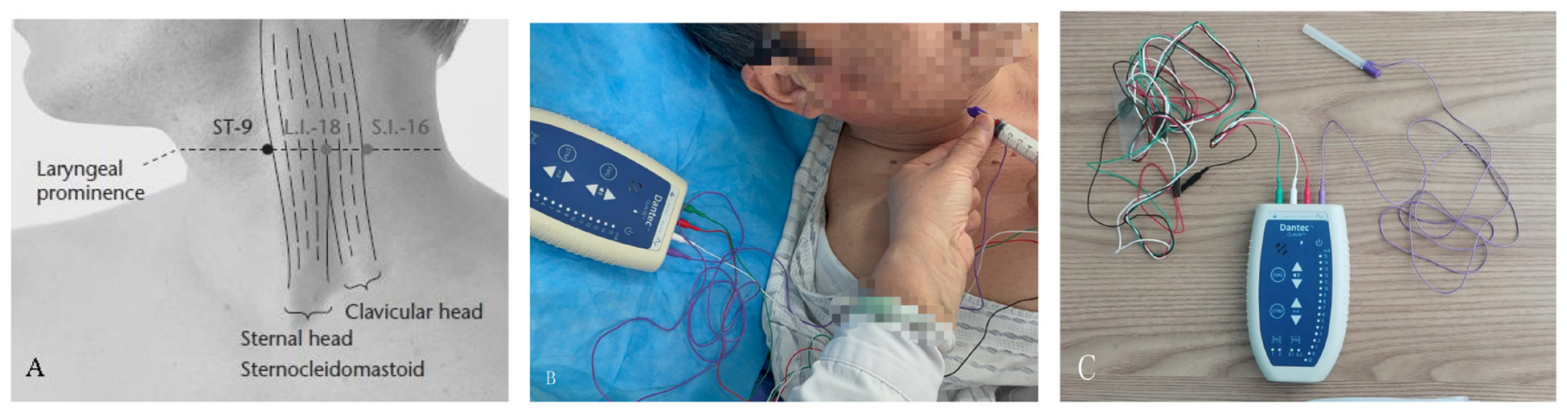
Injection device, anatomical landmarks, and operation schematic. **(A)** Anatomical schematic diagram of the lateral neck, showing key anatomical landmarks: laryngeal prominence, sternocleidomastoid muscle (with its sternal head and clavicular head), and adjacent structures including L.I.-18 and S.I.-16. The injection point (ST-9, Renying acupoint) is located at the level of the laryngeal prominence, at the anterior border of the sternocleidomastoid muscle. It lies lateral to the thyroid cartilage, approximately 1.5 inches from the midline (adjusted individually based on the width of the thyroid cartilage), and is positioned anterior to the common carotid artery, which should be avoided during injection. **(B)** Injection procedure schematic: The patient lies in a supine position with the head slightly tilted backward. The operator uses a dedicated needle electrode equipped with the CLAVIS electromyography device. Under electromyographic guidance, the needle is inserted at a 45° angle inward and upward at the injection site, with a depth of approximately 2/3 of the needle length. When the patient attempts to swallow, the electromyography device displays characteristic motor unit potentials (a “hissing” sound). At this point, switch to stimulation mode to verify the position of the needle tip. After confirming that it is located in the area of the middle pharyngeal constrictor and the thyroarytenoid muscle, slowly inject the medication. **(C)** Photos of main equipment: CLAVIS electromyography main unit, dedicated needle electrode, mecobalamin injection.

Treatment group: In addition to the control group's regimen, the treatment group received EMG-guided targeted injections of mecobalamin into the swallowing muscles. This EMG-guided targeted injection approach for swallowing muscles is consistent with the established methodology for botulinum toxin injection into pharyngeal muscles for dysphagia management, which has been widely validated in previous studies (9). The injection point (ST-9, Renying acupoint) is located at the level of the laryngeal prominence, at the anterior border of the sternocleidomastoid muscle. It lies lateral to the thyroid cartilage, approximately 1.5 inches from the midline (adjusted individually based on the width of the thyroid cartilage), and is positioned anterior to the common carotid artery, which should be avoided during injection (Figure 1A). After routine skin disinfection, 1 mL of mecobalamin injection (SCPC Ouyi Pharmaceutical Co., Ltd., Shijiazhuang, China, NMPA Approval No.: H20055382) was drawn. Using a specialized needle from the CLAVIS electromyograph (Shaanxi Medical Device Approval No. 2014-2250109, NEBN3040) in EMG mode, the needle was inserted slowly near the thyroid cartilage edge, at a 45° angle to the skin, advancing superomedially to about two-thirds of its length. Patients were instructed to attempt a swallowing action. When swallowing activity was detected (as an audible “sizzling” signal), the mode was switched to STIM (output current 5–7 mA, frequency 1 Hz, duration 0.1 s) to confirm needle placement in the middle pharyngeal constrictor and thyroarytenoid muscle region. Then, 0.5 mL of the solution was injected into each side. Real-time EMG monitoring during injection helped avoid major vascular structures. The procedure was repeated every other day. Three sessions constituted one treatment course, with a 2-day interval between courses. All procedures were performed by the same experienced rehabilitation physician. Figures 1A–C illustrates the injection instruments, anatomical landmarks, and procedural schematic.

Both groups underwent WST and VFSS before and 2 weeks after treatment. Assessments were conducted by experienced, uniformly trained therapists blinded to the study design.

### Efficacy evaluation

Efficacy evaluation criteria: (1) WST for dysphagia (10): Patients were graded (1–5) based on their performance swallowing 5 mL and 30 mL of water. Inclusion required a baseline WST score ≥ 3. ① Marked effectiveness: Swallowing function returned to normal (WST grade 1); ② Effectiveness: WST score improved by 1 grade or more but did not reach grade 1; ③ Ineffectiveness: WST score remained unchanged or worsened. (2) VFSS evaluation (11): The Penetration-Aspiration Scale (PAS) was used. Patients ingested thin liquid, thick liquid, and pureed food (mixed with barium sulfate). ① Marked effectiveness: PAS score reduced to 1 (no aspiration or penetration); ② Effectiveness: PAS score reduced by ≥2 points but did not decrease to 1; ③ a reduction of 1 point was considered ineffective; Ineffectiveness: PAS score reduced by less than 2 points, remained unchanged, or worsened.

### Sample size calculation

Based on preliminary pre-test results (unpublished pilot data), the marked effectiveness rate in the treatment group was approximately 50%, and in the control group approximately 20%. Setting α = 0.05 (two-tailed) and power (1–β) = 0.80, using PASS 15.0 software, a sample size of 51 patients per group was required. Considering a 10% dropout rate, 55 patients per group were ultimately included.

### Statistical analysis

All data were analyzed using SPSS 24.0 (SPSS Inc., Chicago, IL, USA). Continuous variables with normal distribution were expressed as mean ± standard deviation, and between-group comparisons were performed using independent samples *t*-tests. Non-normally distributed data were expressed as median (interquartile range), and between-group comparisons were performed using the Mann-Whitney U-test. Categorical data were expressed as frequency (percentage), and between-group comparisons were performed using the chi-square (χ^2^) test or Fisher's exact test (when expected frequency < 5). Within-group comparisons of pre- and post-treatment outcomes were performed using paired *t*-tests for normally distributed data or Wilcoxon signed-rank tests for non-normally distributed data. A *P-value* < 0.05 was considered statistically significant. Test statistics (e.g., *t*-value, χ^2^-value, Z-value), degrees of freedom, and effect sizes (e.g., Cohen's d) were reported where appropriate.

## Results

### Comparison of baseline characteristics

The treatment group consisted of 29 males and 26 females, aged 35–69 years (mean 62 ± 5 years), with disease duration ranging from 3 to 22 days (mean 16.6 ± 5.1 days). The control group included 30 males and 25 females, aged 33–70 years (mean 65 ± 7 years), with disease duration ranging from 4 to 23 days (mean 14.7 ± 4.9 days). Although the between-group differences in age and disease duration did not reach the conventional statistical significance threshold (*P* < 0.05), they showed a trend toward marginal significance (*P* < 0.08). However, these differences were not clinically meaningful, as the age ranges and disease durations of both groups fell within the typical spectrum of patients with post-medullary infarction dysphagia, and the effect sizes for these differences were small (Cohen's *d* < 0.3), indicating minimal impact on the study outcomes. No significant differences were observed between the two groups regarding gender, pre-treatment WST grade, or VFSS PAS scores (all *P* > 0.5), and the pre-treatment aspiration incidence of different food consistencies was also comparable between groups (*P* > 0.05), confirming overall baseline comparability (Table 1).

**Table 1:** Baseline characteristics of patients in both groups.

Characteristic	Treatment group (*n* = 55)	Control group (*n* = 55)	Test statistic	*P-value*
Age (years, mean ± SD)	62.3 ± 5.2	64.8 ± 6.9	*t* = 1.83	0.07
Gender (Male/Female)	29/26	30/25	*χ^2^* = 0.04	0.85
Disease Duration (days, mean ± SD)	16.6 ± 5.1	14.7 ± 4.9	*t* = 1.91	0.06
Pre-treatment WST Grade [Median (IQR)]	4 (3–4)	4 (3–4)	*Z* = −0.45	0.65
Pre-treatment VFSS PAS Score [Median (IQR)]	6 (5–7)	6 (5– 7)	*Z* = −0.32	0.75
Pre-treatment thin liquid aspiration [*n* (%)]	50 (90.9)	49 (89.1)	*χ^2^* = 0.12	0.73
Pre-treatment thick liquid aspiration [*n* (%)]	45 (81.8)	46 (83.6)	*χ^2^* = 0.06	0.81
Pre-treatment puree aspiration [*n* (%)]	38 (69.1)	36 (65.5)	*χ^2^* = 0.17	0.68

SD, Standard Deviation; IQR, Interquartile Range; WST, Wada Water Swallowing Test; VFSS, Videofluoroscopic Swallowing Study; PAS, Penetration-Aspiration Scale.

### Comparison of WST efficacy

After treatment, the marked effectiveness rate and total effectiveness rate in the treatment group were significantly higher than those in the control group (*P* < 0.05, Table 2). The total effectiveness rate was 94.6% in the treatment group and 85.5% in the control group (χ^2^ = 4.27, *df* = 1, *P* = 0.039). The marked effectiveness rate was 47.3% in the treatment group and 16.4% in the control group (χ^2^ = 12.47, *df* = 1, *P* < 0.001).

**Table 2:** Comparison of WST efficacy between the two groups [*n* (%)].

Group	*N*	Marked effectiveness	Effective	Ineffective
Treatment group	55	26 (47.3)	26 (47.3)	3 (5.4)
Control group	55	9 (16.4)	38 (69.1)	8 (14.5)
*χ^2^*		12.11	5.38	2.53
*P-value*		0.001	0.20	0.11

### Comparison of VFSS efficacy

After treatment, the marked effectiveness rate and total effectiveness rate in the treatment group were significantly higher than those in the control group (*P* < 0.05, Table 3). The total effectiveness rate was 96.4% in the treatment group and 83.6% in the control group (χ^2^ = 4.95, *df* = 1, *P* = 0.026). The marked effectiveness rate was 50.9% in the treatment group and 18.2% in the control group (χ^2^ = 13.09, *df* = 1, *P* < 0.001).

**Table 3:** Comparison of VFSS efficacy between the two groups [*n* (%)].

Group	*N*	Marked effectiveness	Effective	Ineffective
Treatment group	55	28 (50.9)	25 (45.5)	2 (3.6)
Control group	55	10 (18.2)	36 (65.4)	9 (16.4)
*χ^2^*		13.03	4.45	4.95
*P-value*		< 0.001	0.04	0.03

### Comparison of aspiration incidence

Within-group comparison: Compared with before treatment, the incidence of aspiration during ingestion of thin liquid, thick liquid, and pureed food decreased significantly in both groups after treatment (*P* < 0.05). Between-group comparison: After treatment, the incidence of aspiration in the treatment group was significantly lower than that in the control group for all three food consistencies (*P* < 0.05, Table 4).

**Table 4:** Comparison of aspiration incidence during VFSS before and after treatment [*n* (%)].

Food consistency	Treatment group (***n*** = 55)		Control group (***n*** = 55)	
	Before treatment	After treatment	Before treatment	After treatment
Thin liquid	50 (90.9)	20 (40.0)^*^	49 (89.1)	35 (71.4)^*, Δ^
Thick liquid	45 (81.8)	15 (33.3)^*^	46 (83.6)	36 (78.3)^*, Δ^
Puree	38 (69.1)	8 (21.1)^*^	36 (65.5)	20 (55.6)^*, Δ^

^*^P < 0.05 compared with before treatment within the same group (McNemar's test).

ΔP < 0.05 compared between-group post-treatment.

### Adverse events

No serious adverse events such as hematoma, infection, or nerve injury occurred in this study. Minor adverse reactions like injection site pain (3 cases in the treatment group) resolved spontaneously without specific management.

## Discussion

Dysphagia caused by medullary paralysis is a common and challenging neurogenic condition in stroke survivors, primarily affecting the pharyngeal phase with a high incidence (12, 13). Aspiration is its most frequent and serious manifestation (14, 15). Conventional treatments like rehabilitative training, acupuncture, and neurostimulation often have slow onset and suboptimal efficacy (4).

Medullary injury causes pharyngeal constrictor muscle paresis, leading to weak contractions and impaired bolus propulsion (16). The brainstem swallowing centers, located in the dorsal and ventrolateral medulla, control reflexive swallowing, and peripheral sensory feedback continuously modulates these centers (1). After central damage, swallowing stimuli can promote functional reorganization by recruiting other neurons (17). In this study, EMG guidance ensured precise localization of mecobalamin injection into the neuromuscular junctions. Mecobalamin exerts local neurotrophic effects by promoting nerve cell metabolism, myelination, and synaptic development, thereby facilitating axonal regeneration and motor recovery (6). Research also indicates that mecobalamin promotes synaptic growth and inhibits neuronal apoptosis (18). The complex anatomy of the region demands precision; combining EMG guidance with targeted injection enhances both safety and accuracy. The absence of serious adverse events in this study supports the safety of this procedure when performed by experienced clinicians following the described protocol.

Our method synergizes targeted neurotrophic drug delivery with EMG guidance, aiming for rapid recovery of paralyzed muscles. Using VFSS, the gold standard for swallowing assessment, we objectively evaluated treatment effects. The results demonstrate that adjunctive EMG-guided mecobalamin injection significantly improves swallowing efficacy and reduces aspiration risk compared to conventional therapy alone. The significant within-group improvements in both groups also underscore the value of conventional rehabilitation.

This study has certain limitations. First, the sample was drawn from a single center, which may affect the generalizability of the findings. Second, the study only evaluated short-term effectiveness at 2 weeks; long-term outcomes remain unknown. Third, while we used objective outcome measures, assessor blinding helped mitigate bias, but a double-blind design was not feasible due to the nature of the intervention. Future multicenter studies with larger cohorts, longer follow-up durations, and sham-controlled designs are warranted to validate these results and assess the long-term efficacy and safety of the intervention.

## Conclusions

In conclusion, electromyography-guided targeted injection of mecobalamin, as an adjunct to conventional therapy, appears to be a rapid and effective treatment strategy for dysphagia following medullary paralysis, yielding significantly higher marked effectiveness rates compared to rehabilitation training alone.

## Data Availability

The original contributions presented in the study are included in the article/supplementary material, further inquiries can be directed to the corresponding author.
